# Spinal Cord Infarction Versus Idiopathic Transverse Myelitis: Clinical, Radiological, and Functional Insights From a Retrospective Cohort Study

**DOI:** 10.1002/acn3.70312

**Published:** 2026-01-27

**Authors:** Zeqiang Ji, Jianlong Zhang, Yiming Shi, Shiyu Shan, Yang Du, Guangshuo Li, Ying Jin, Yani Zhang, Chuanying Wang, Yijun Lin, Yuhao Guo, Decai Tian, Xingquan Zhao, Tian Song

**Affiliations:** ^1^ Department of Neurology Beijing Tiantan Hospital, Capital Medical University Beijing China; ^2^ China National Clinical Research Center for Neurological Diseases Beijing China; ^3^ Department of Neurology Tianjin Neurological Institute, Tianjin Medical University General Hospital Tianjin China; ^4^ Department of Neurology The First Affiliated Hospital of Xinxiang Medical University Xinxiang China; ^5^ Department of Radiology Beijing Tiantan Hospital, Capital Medical University Beijing China; ^6^ Department of Internal Medicine MedStar Washington Hospital Center Washington DC USA; ^7^ Department of Medicine Danbury Hospital/Zucker School of Medicine at Hofstra/Northwell Danbury Connecticut USA

**Keywords:** differential diagnosis, idiopathic transverse myelititis, spinal cord infarction

## Abstract

**Introduction:**

Spinal cord infarction (SCI) is a rare but devastating myelopathy, characterized by a high disability rate and an unfavorable prognosis. It has often been underdiagnosed and misdiagnosed as idiopathic transverse myelitis (ITM). This study aimed to describe the clinical features, radiological biomarkers, treatments, and functional outcome of SCI, distinguishing it from ITM.

**Methods:**

A retrospective observational cohort study included patients who met the diagnostic criteria of SCI and ITM from January 2019 to October 2024. The clinical, radiological data, and diagnosis were recorded, and the functional outcomes were reached via telephone and face‐to‐face evaluations. Univariate analysis was used to differentiate the two groups.

**Results:**

During the study period, a total of 22 SCI patients with a median age of 53.0 years (interquartile range (IQR): 41.8 to 60.2) were enrolled. Thirteen patients underwent the diffusion‐weighted imaging (DWI) and the apparent diffusion coefficient (ADC) sequence, among whom 12 were confirmed as having definite SCI. Compared with ITM, SCI has the following characteristics. The time from onset to nadir in SCI is much shorter, mostly within 6 h (*p* < 0.001). On sagittal MRI, SCI often manifests as linear lesions, while ITM tends to present as patchy and fusiform lesions (*p* < 0.001). We have also defined a lesion characteristic of SCI based on T2‐weighted sequences, termed the “eccentric sign”. Moreover, patients with SCI generally have a poorer prognosis and higher dependence.

**Conclusions:**

SCI can be diagnosed and differentiated from ITM based on clinical features and radiological signs.

## 1 Introduction

Spinal cord infarction (SCI) is a rare form of myelopathy that accounts for nearly 1.0% of all stroke cases and 6% of all acute myelopathies, with a high mortality rate and unfavorable prognosis [1, 2, 3]. SCI usually manifests as deficits of the motor, sensory, and autonomic nervous systems [4]. Compared to cerebral infarction, SCI generally manifests at an earlier stage of life, leading to a greater disease burden and a diminished life expectancy [5]. However, due to the lack of definitive diagnostic criteria, the diagnosis of SCI is generally made clinically, with radiological signs to validate the diagnosis and exclude other etiologies. Meanwhile, the underlying cause of spontaneous SCI remains incompletely understood [6]. However, the underutilization of spinal cord MRI with DWI/ADC sequences, coupled with the difficulty in identifying the characteristic signs of SCI, leads to approximately 15% of SCI cases being initially misdiagnosed as idiopathic transverse myelitis (ITM) [7, 8]. This underscores the criticality of distinguishing between SCI and ITM, which is essential for the subsequent treatment and secondary prevention of SCI. Furthermore, there is limited evidence regarding the treatment of SCI, particularly concerning the use and effectiveness of corticosteroids and antiplatelet agents [6]. Due to the limited studies, we consecutively enrolled patients with SCI and ITM, comparing their clinical characteristics, imaging findings, treatments, and prognosis, aiming to provide references for subsequent diagnosis and treatment strategies.

## 2 Methods

### 2.1 Patients and Diagnostic Criteria

We retrospectively enrolled patients admitted to Beijing Tiantan Hospital from January 2019 to October 2024 who were diagnosed with spontaneous spinal cord infarction (SCI) and idiopathic transverse myelitis (ITM) via searching the Electronic Medical Record (EMR) system. All patients underwent conventional spinal magnetic resonance imaging (MRI) and cerebrospinal fluid tests. To facilitate a more accurate comparison between SCI and ITM, only definite and probable SCI are enrolled to prevent selection bias. The diagnostic criteria for SCI were by the diagnostic criteria published by Zalewski et al. [4]. The inclusion criteria of our study include (1) final diagnosis of spontaneous SCI recorded on EMR; (2) adequate clinical and radiologic data (consistent with SCI and/or rule out alternative etiologies) and the exclusion criteria included (1) incomplete medical record; (2) other confirmed etiologies such as compressive myelopathy, autoimmune, infectious myelitis or systemic disease.

The diagnosis for ITM followed the diagnostic criteria established by the Transverse Myelitis Consortium Working Group in 2002 [9]. The inclusion criteria contained (1) bilateral symptoms/signs of spinal cord dysfunction evolving over 4 h to 21 days; (2) a sensory level on the trunk and evidence of inflammation (MRI gadolinium enhancement or CSF evidence), and the exclusion criteria included (1) incomplete medical record; (2) confirmed other etiologies (compressive myelopathy, autoimmune, infectious myelitis, or systemic disease). This study was conducted in accordance with the Declaration of Helsinki World Medical Association and was approved by the Institutional Review Committee (IRB) of Beijing Tiantan Hospital (KY‐2021‐150‐01).

### 2.2 Clinical Variables

To identify the clinical features of SCI and ITM, we collected demographics, medical history, motor, sensory, and autonomic dysfunction at nadir and evaluated the time to nadir at admission. We also recorded the immunotherapy, such as corticosteroid and gamma‐immunoglobulin, as well as the antiplatelet, anticoagulant, lipid‐lowering therapy, and vitamin use after onset.

### 2.3 Laboratory Evaluation

Laboratory evaluation variables include the vitamin B12, B6 and folate; bacterial and virology tests (syphilis serology, Lyme disease serology, herpes simplex virus and varicella‐zoster virus serology, HIV serology, human T‐lymphotropic virus‐1 serology), autoimmune antibodies (antinuclear antibody, anti–cyclic citrullinated peptide, anti–neutrophil cytoplasmic antibody, antiphospholipid antibody), demyelination antibodies (aquaporin‐4–IgG, myelin oligodendrocyte glycoprotein–IgG, glial fibrillary acidic protein–IgG, myelin basic protein–IgG, cell‐based assay (CBA)), and paraneoplastic autoantibody evaluation. None of those tests revealed a positive result attributed to a specific etiology.

### 2.4 Cerebrospinal Fluid Evaluation

Cerebrospinal fluid evaluations included open pressure, whole blood cell count, protein, chloride, and glucose. Additional CSF testing included oligo‐clonal bands, cytology, flow cytometry, Gram stain, bacterial culture, venereal disease research laboratory, Lyme disease serology, and polymerase chain reaction (PCR), Cryptococci antigen, angiotensin‐converting enzyme, paraneoplastic autoantibody evaluation, varicella‐zoster virus PCR, Epstein–Barr virus PCR, cytomegalovirus PCR, enterovirus PCR, *mycobacterium tuberculosis* PCR, and culture. All SCI and ITM cohorts underwent screening for demyelinating antibodies—namely, aquaporin‐4–IgG, myelin oligodendrocyte glycoprotein–IgG (MOG‐IgG), glial fibrillary acidic protein–IgG (GFAP‐IgG), and myelin basic protein–IgG (MBP‐IgG)—using cell‐based assays (CBA).

### 2.5 Neuroimaging

The neuroimaging was performed with a 3.0 Tesla MRI scanner (General Electric, Discovery MR750). Sagittal spinal cord T2‐weighted spin‐echo images were acquired with time of repetition (TR) 1800–4300 ms, time of echo (TE) 80–130 ms, in‐plane resolution of 0.35–1 mm, and a section thickness of 3–4.5 mm. The Gadolinium enhancement MRI (Gd‐MRI) was also administered with a TR 100–1300 ms, TE 3–15 ms, and identical in‐plane resolution and slice thickness as the T2W images. Also, part of the suspected SCI patients underwent the MRI diffusion‐weighted imaging (DWI) and apparent diffusion coefficient (ADC) sequence.

The specified SCI signs, like owl eyes [10] sign and pencil‐like hyperdensities [11], were interpreted according to their definitions. The computed tomography angiography (CTA), MR angiography (MRI), or digital subtraction angiography (DSA) was performed to rule out the spinovascular malformation, fistula, or aneurysm. Some of the MRIs were done in the early phase in other medical institutions, which were also interpreted and recorded. All the image interpretations were conducted by two experienced radiologists and supervised by all neurologists.

### 2.6 Follow‐Up Analysis

We evaluated the modified Rankin Scale (mRS), the American Spinal Injury Association Impairment Scale [12], the use of gait aids, canes, wheelchairs, and urinary catheters. All the follow‐up was done via cellphone or face‐to‐face (Figure S1).

### 2.7 Statistics Analysis

Continuous variables are expressed as medians (interquartile range, IQR) or means ± standard deviation (SD) and were evaluated with the Shapiro–Wilk test and compared using the Mann–Whitney or *t* test, respectively. The chi‐square test or Fisher's exact test were used for the comparison of categorical variables, which are expressed as numbers (proportions). To effectively control the type I error that may be caused by multiple tests, we employed the False Discovery Rate (FDR) control method to obtain the corrected *p* value (i.e., *q* value). Only when *q* < 0.05 were the related diagnostic features considered significant. The results were considered significant for *p* values < 0.05 (two‐sided).

## 3 Results

### 3.1 Demographics and Clinical Features, Diagnosis, and Prognosis

A total of 22 SCI patients were included, with a median age of 53.0 years (range: 24–71 years). The selection process was demonstrated in Figure S1. There were 11 males (50.0%). 11 patients (50.0%) had at least one vascular disorder risk factor. The admission median mRS in SCI cohort was 4.0 (IQR: 3.0 to 5.0), indicating a more severe disability status compared to ITM (4.0 vs. 2.0, *p* = 0.021).

The SCI patients typically experienced a much shorter time from symptom onset to nadir, with the majority (16 patients, 72.7%) reaching peak severity within 6 h. Two patients reached nadir between 6 and 12 h, one between 12 and 24 h, and three patients took over 24 h to reach nadir. This was significantly shorter than the time observed in ITM patients (*p* < 0.001).

All the SCI patients presented with limb weakness, mainly bilaterally (90.9%), and 11 of them experienced paraplegia. The majority of 17 of them had bilateral sensory loss and 15 (68.2%) patients had sensory loss level. 11 patients had neck or back pain soon after the event, and 18 (81.8%) patients had bladder or bowel dysfunction.

In comparison to the SCI cohort, the ITM group exhibited significantly milder clinical functional deficits with a median mRS of 2 (IQR: 1 to 3, *p* < 0.05). ITM patients had a longer time to nadir, with 35 patients (85.4%) reaching their nadir over 24 h; 11 (26.8%) presented with neck and back pain. ITM was also presented with paraplegia, but at a lower prevalence compared with the SCI patients. In sensory loss, the ITM had more superficial sensory loss and less sensory loss level than SCI patients (Table 1).

**TABLE 1:** Baseline characteristics of SCI and ITM.

Clinical characteristics	Spinal cord infarction, *N* = 22	Idiopathic transverse myelitis, *N* = 41	*p*
Male, *n* (%)	11 (50.0)	26 (63.4)	0.446
Age, years	53.0 [41.8, 60.2]	49.0 [36.0, 59.0]	0.564
Smoking, *n* (%)	7 (31.8)	14 (34.1)	1
Hypertension, *n* (%)	4 (18.2)	11 (26.8)	0.647
Diabetes, *n* (%)	3 (13.6)	5 (12.2)	1
Hyperlipidemia, *n* (%)	3 (13.6)	0 (0.0)	0.068
Provoking factors, *n* (%)			0.311
None	18 (81.8)	32 (78.0)	
Infection	2 (9.1)	8 (19.5)	
Labor	2 (9.1)	1 (2.4)	
Admission mRS	4.0 [3.0, 5.0]	2.0 [1.0, 3.0]	0.021
Rapid onset to nadir, *n* (%)	19 (86.4)	4 (9.8)	< 0.001
Attack frequency	1.0 [1.0, 2.0]	1.0 [1.0, 1.0]	0.037
Time to nadir deficit, *n* (%)			< 0.001
≤ 6 h	16 (72.7)	0 (0.0)	
6–12 h	2 (9.1)	6 (14.6)	
12–24 h	1 (4.5)	0 (0.0)	
> 24 h	3 (13.6)	35 (85.4)	
Associated pain, *n* (%)	11 (50.0)	11 (26.8)	0.118
Bowel/bladder dysfunction, *n* (%)	18 (81.8)	24 (58.5)	0.112
Respiratory failure, *n* (%)	2 (9.1)	0 (0.0)	0.118
Limb palsy, *n* (%)			< 0.001
None	0 (0.0)	24 (58.5)	
Quadriplegia	8 (36.4)	1 (2.4)	
Paraplegia	11 (50.0)	12 (29.3)	
Hemiplegia	3 (13.6)	4 (9.8)	
Bilateral sensory loss, *n* (%)	17 (77.3)	27 (65.9)	0.182
Selective sensory loss, *n* (%)	4 (18.2)	0 (0.0)	0.023
Sensory level, *n* (%)	15 (68.2)	6 (14.6)	< 0.001
Bilateral weakness, *n* (%)	20 (90.9)	15 (36.6)	< 0.001
Absent reflexes/flaccid tone, *n* (%)	7 (31.8)	3 (7.3)	0.03
Babinski sign, *n* (%)	9 (40.9)	18 (43.9)	1
*Radiology findings*			
Onset to T2, days	4.5 [2.0, 11.50]	33.0 [18.0, 50.0]	< 0.001
Spinal level, *n* (%)			0.227
Cervical	6 (27.3)	21 (51.2)	
Thoracic	9 (40.9)	14 (34.1)	
Lumbar	1 (4.5)	0 (0.0)	
Cervical+ Thoracic	4 (18.2)	5 (12.2)	
Thoracic+ Lumbar	2 (9.1)	1 (2.4)	
DWI/ADC restriction, *n* (%)	12 (92.3)	—	—
*T2‐hyperintensity patterns*			
Owl eyes, *n* (%)	6 (27.3)	0 (0.0)	0.001
Anterior pencil‐like hyperintensity, *n* (%)	14 (63.6)	0 (0.0)	< 0.001
Anterior U/V, *n* (%)	1 (4.5)	0 (0.0)	0.118
Anteromedial spot, *n* (%)	8 (36.4)	0 (0.0)	< 0.001
Holocord, *n* (%)	2 (9.1)	0 (0.0)	0.349
Hologrey, *n* (%)	1 (4.5)	0 (0.0)	0.118
The eccentric sign, *n* (%)	17 (77.3)	4 (9.8)	< 0.001
Concurrent acute cerebral infarct, *n* (%)	1 (4.5)	1 (2.4)	1
With white matter lesions, *n* (%)	3 (13.6)	22 (53.7)	0.005
Patchy lesions*, *n* (%)	3 (13.6)	35 (85.4)	< 0.001
Lesion length, mm	72.0 ± 12.4	62.6 ± 10.7	0.254
Segment invloved	3.0 [2.8, 3.5]	3.0 [2.0, 3.3]	0.137
Onset to Gd‐MRI, days	6.0 [2.0, 15.0]	33.0 [18.0, 50.0]	< 0.001
Gadolinium enhancement, *n* (%)	19 (86.4)	34 (82.9)	1
*CSF findings*			
Open pressure, mmH_2_O	150 [126, 171]	135 [115, 160]	0.414
Oligoclonal bands	4 (18.2)	14 (34.1)	0.296
Total cell, cells/mm^3^	4.0 [2.0, 10.8]	15.0 [5.0, 105.0]	0.019
Total protein, mg/dL	46.15 [38.4, 121.8]	40.35 [31.2, 45.4]	0.053
Glucose, mmol/L	4.14 [3.5, 4.5]	3.8 [3.4, 4.2]	0.166
Chlorides, mmol/L	124 [123, 127]	126 [125, 128]	0.1
*Treatments*			
Corticosteroids, *n* (%)	20 (90.9)	38 (92.7)	0.698
γ‐Immunoglobin, *n* (%)	9 (40.9)	8 (19.5)	0.051
Antiplatelet use, *n* (%)	15 (68.2)	0 (0.0)	< 0.001
Anticoagulant use, *n* (%)	3 (13.6)	0 (0.0)	0.068
Lipid‐lowering drug use, *n* (%)	5 (22.7)	3 (7.3)	0.067
Vitamin use, *n* (%)	21 (95.5)	29 (70.7)	0.006

Abbreviations: CSF, cerebrospinal fluid; DWI/ADC, diffusion‐weighted imaging/apparent diffusion coefficient; Gd‐MRI, gadolinium enhancement magnetic resonance imaging; ITM, idiopathic transverse myelitis; MRI, magnetic resonance imaging; mRS, modified Rankin Scale; SCI, spinal cord infarction. *:If the patients' lesions were not patchy or spindle‐like lesions, it was the linear lesion.

### 3.2 Cerebrospinal Fluid Tests

All the SCI (*n* = 22) and ITM (*n* = 41) patients completed lumbar puncture within the first week after admission. None had results consistent with a specific infection, demyelination, autoantibody, neoplastic, paraneoplastic, or other etiology. The ITM group had higher CSF cell count (15.0/mL (IQR: 5.0, 105.0) versus 4.0 (IQR: 2.0, 10.8), *p* = 0.019) than SCI (Table 1).

### 3.3 The Neuroimaging Characteristics

Spinal sagittal and axial MRI, as well as spinal gadolinium enhancement MRI, were performed in all patients from both cohorts.

In the SCI cohort, the median time from onset to the initial MRI was 4.5 days (IQR: 2.0 to 11.5 days). Among the patients, 13 underwent DWI/ADC imaging, and 12 of these demonstrated restricted diffusion. The median time from symptom onset to DWI/ADC imaging was 13.0 days (IQR: 11.3 to 14.5 days).

Regarding the involved spinal segments, the cervical and the thoracic level were the most invaded levels, the specific signs of SCI like the owl eyes sign, anterior pencil‐like hyperintensity, anterior U/V, anteromedial spot, holocord, and hologrey were 6 (27.3%), 14 (63.6%), 1 (4.5%), 8 (36.4%), 2 (9.1%), and 1 (4.5%), respectively. Notably, the median first T2 scan time was 4.5 days (IQR 2–11.5 days); 16/22 were acquired ≤ 7 days and 6/22 at 8–14 days. Per Zalewski criteria [4] (acute ≤ 7 days; subacute 7–28 days), 65% were documented in the acute phase and 35% in the early‐subacute phase; per Leys criteria [13] (> 6 h—a few days = acute; a few days—weeks = subacute), approximately half occurred in the acute stage and half in the subacute stage (Table S1). Ninteen patients (86.3%) were observed to have gadolinium enhancement. Two patients demonstrated vertebral artery dissection, confirmed by CTA and DSA (seen in Table S1).

**FIGURE 1:**
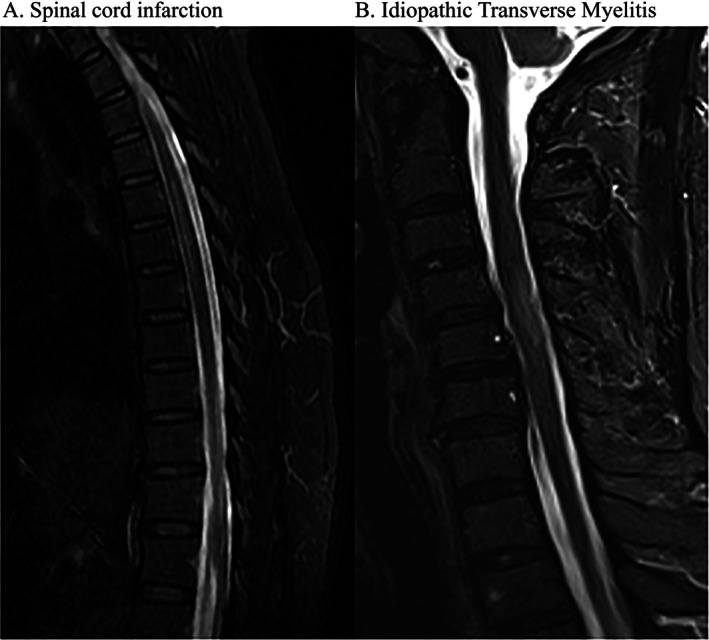
The linear lesion in SCI versus the patchy lesion in ITM on sagittal spinal MRI. (A) The linear lesion (red arrow) depicted on the sagittal spinal MRI of a patient (Case 11) diagnosed with definite spinal cord infarction. (B) The patchy lesion (red arrow) depicted on the sagittal spinal MRI of a patient (Case 9) diagnosed with ITM. ITM, idiopathic transverse myelitis; MRI, magnetic resonance imaging; SCI, spinal cord infarction.

Considering that several characteristic signs of SCI may be subject to complexities such as conceptual intricacy and detection rates limited by the time interval from disease onset to imaging, we propose a relatively simple differential diagnosis method. We simply classified the lesions on sagittal MRI into linear or patchy/spindle‐shaped categories. We found that SCI was more commonly associated with linear lesions. In contrast, ITM typically presented with patchy or spindle‐shaped lesions (19 (86.4%) linear lesions in SCI vs. 35 (85.4%) patchy or spindle‐shaped lesions in ITM, *p* < 0.001) (seen in Figure 1).

**FIGURE 2:**
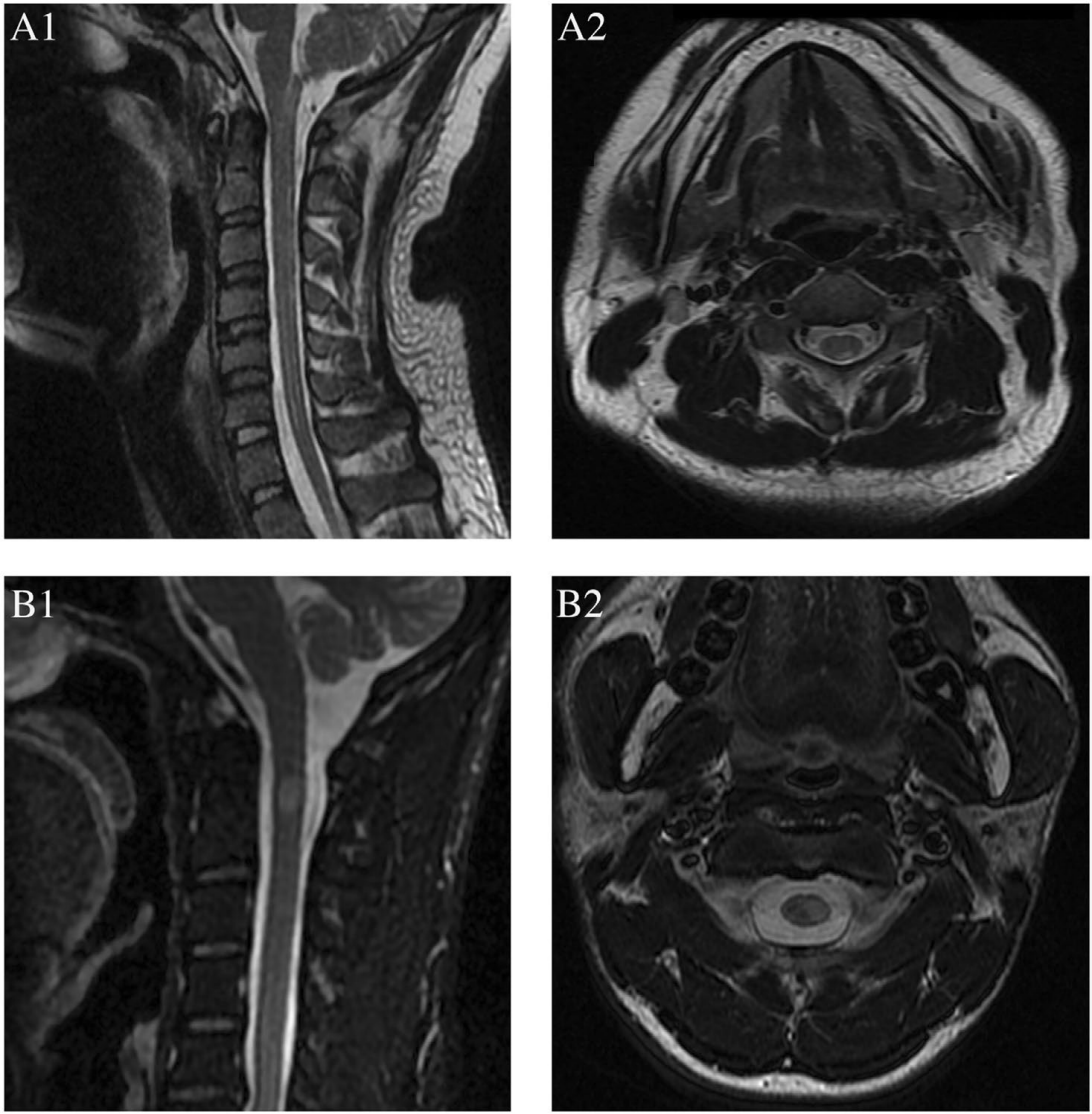
The eccentric signs of SCI on axial spinal MRI. The eccentric signs of SCI of a SCI patient on the sagittal (A1) and the axial (A2) spinal MRI are compared with an ITM patient (B1–B2). ITM, idiopathic transverse myelitis; MRI, magnetic resonance imaging; SCI, spinal cord infarction.

Additionally, we observed that in SCI patients confirmed by DWI/ADC, the MRI findings often exhibit specific characteristics. The lesions are typically located in the vascular distribution areas and are usually situated in the peripheral part of the spinal cord plane, not centrally, but peripherally located in the spinal cord; the central axis of the lesion is not coaxial with the spinal cord (Figure 2). We define this phenomenon as the “eccentric sign”, which is frequently observed in SCI but not in ITM, as seen in Figure 2.

We also observed that the prevalence of concurrent white matter lesions (WML) was higher in ITM patients than in SCI patients (22 (53.7%) vs. 3 (13.6%), *p* = 0.005), which suggests that ITM and WML may share certain etiological similarities (Table 1).

We also concluded and compared the radiological characteristics of different etiologies (VAD and idiopathic with atherosclerotic risk factors) in Table S2.

All the clinical and radiological biomarkers were corrected by the False Discovery Rate (FDR) control method and seen in Table S3.

### 3.4 The Diagnosis of the SCI Group

By the diagnostic criteria published by Zalewski et al. and confirmed by 2 experienced myelopathy neurologists (TS and DT), 12 patients were diagnosed as definite SCI and 10 patients were diagnosed as probable SCI.

### 3.5 The Treatment

In SCI patients, the immunotherapy was conducted at an early disease stage for symptom relief. The corticosteroids were used in 20 (90.9%) cases, with 15 (75.0%) cases reporting no significant relief. Intravenous immunoglobulin was used in 9 (40.9%) cases. In terms of the secondary prevention of spinovascular disease, antiplatelet treatment with at least 1 agent, 15 (68.2%); anticoagulant treatment, 3 (13.6%); antilipidemia treatment, 5 (22.7%). 4 patients did not receive the antithrombotic/antiplatelet due to their high risk of bleeding (gastric bleeding history: 2 cases) and refusal (2 cases). None underwent the monoclonal antibody treatment, thrombolysis or spinal depression surgery.

In ITM cohort, 38 (92.7%) patients underwent corticosteroids, and 8 (19.5%) received immunoglobulin. Notably, 35 patients reported significant relief during or after the immunotherapy. No antiplatelet or anticoagulant therapy was reported.

### 3.6 The Follow‐Up Clinical Outcomes

The follow‐up clinical outcomes were acquired from 19 patients (86.4%) in SCI and 29 (70.7%) in ITM. In SCI patients, the median follow‐up time was 8.0 months (6.0 to 10.3). The median mRS at last follow‐up was 3.0 (3.0, 4.0). The American Spinal Injury Association Impairment Scale (ASIA score) outcomes were graded as follows: A: 0 (0.0%); B: 2 (10.5%); C: 2 (10.5%); D: 11 (57.9%); E: 3 (15.8%). Ambulatory outcome at last follow‐up was: no gait aid, 7 (36.8%); cane, 8 (42.1%); wheelchair, 5 (26.3%); pain: 4 (21.1%); urinary catheter: 3 (15.8%); and no death. No patients developed recurrent episodes of SCI during follow‐up.

In comparison to the SCI, ITM patients showed more favorable clinical outcomes in mRS and ASIA grades (Table 2).

**TABLE 2:** Clinical outcomes of followed‐up SCI and ITM.

Clinical characteristics	Spinal cord infarction, *N* = 19	Idiopathic transverse myelitis, *N* = 29	*p*
Follow‐up time, months	8.0 (6.0, 10.3)	22.0 (12.0, 32.5)	< 0.01
mRS, *n* (%)			< 0.01
0	0 (0.0)	0 (0.0)	
1	0 (0.0)	15 (51.7)	
2	3 (15.8)	10 (34.5)	
3	11 (57.9)	4 (13.8)	
4	3 (15.8)	0 (0.0)	
5	1 (5.3)	0 (0.0)	
6	0 (0.0)	0 (0.0)	
ASIA grade, *n* (%)			< 0.01
A	0 (0.0)	0 (0.0)	
B	2 (10.5)	0 (0.0)	
C	2 (10.5)	0 (0.0)	
D	11 (57.9)	4 (13.8)	
E	3 (15.8)	25 (86.2)	
No gait aid, *n* (%)	7 (36.8)	26 (89.7%)	0.013
Wheelchair, *n* (%)	5 (26.3)	0 (0.0)	0.04
Cane use, *n* (%)	8 (42.1)	3 (10.3)	0.007
Urinary catheter, *n* (%)	3 (15.8)	0 (0.0)	0.068
Pain, *n* (%)	4 (21.1)	0 (0.0)	0.022

Abbreviations: ITM, idiopathic transverse myelitis; mRS, modified Rankin Scale; SCI, spinal cord infarction.

## 4 Discussion

Spinal cord infarction is an uncommon vascular and spinal cord disorder. Owing to the lack of definite diagnostic criteria, SCI is frequently underdiagnosed and misdiagnosed as ITM over an extended period. To the best of our knowledge, this study is the first to illustrate the distinct MRI characteristics of the SCI and to differentiate it from the ITM. We have also compared the clinical and radiological features, treatment, and prognoses of patients of two cohorts, aiming to provide valuable information for differential diagnosis.

Our research findings indicate that the time to nadir in SCI is significantly shorter than that in ITM, typically occurring within less than 6 h, which is consistent with previous studies [14]. The shorter onset time of SCI compared to ITM can be attributed to the acute nature of vascular occlusion versus the more gradual inflammatory process in ITM. In SCI, the sudden blockage of a spinal artery, such as the anterior spinal artery, leads to rapid ischemia and subsequent tissue damage. The most notable clinical feature of SCI is pain, accompanied by more severe limb weakness, more frequent bilateral weakness, obvious sensory level damage, and more acute ANS symptoms. Clinical symptoms are the primary basis for the early detection of spinal cord injury [4, 14].

We observed that over 70% of SCI patients exhibited linear lesions on sagittal T2‐weighted images, whereas ITM primarily manifested as patchy or fusiform lesions with a more diffuse distribution. This discrepancy may be attributed to the fact that in SCI, ischemic injury results from the occlusion of spinal cord vasculature, leading to insufficient perfusion of downstream penetrating arteries. Given that these arteries typically have a longitudinal distribution. Therefore, the linear changes, or so‐called long segment lesions [4], are depicted on sagittal MRI.

In the axial plane, aside from the highly specific “owl eyes” sign [15] resulting from symmetrical damage due to anterior spinal artery (ASA) occlusion [16]. The eccentric distribution of SCI lesions, as described in our study, may stem from the occlusion of specific perforating arteries. An infarct in the posterolateral spinal cord (near the surface) could affect regions supplied by the posterior spinal artery or radicular arteries, leading to involvement of the posterior horn or lateral column. Alternatively, this pattern may reflect the compromised blood supply to the peripheral white matter regions of the spinal cord (e.g., posterior or lateral columns), which are more vulnerable to hypoperfusion or embolic events. Unlike SCI, ITM lesions are driven by an inflammatory process without a clear relationship to vascular distribution.

We also observed a greater percentage of enhancement during the initial stage of spinal cord infarction, which contradicts the earlier assertion that enhancement indicates ITM or other factors [11]. 86% of patients underwent the Gd‐MRI between 3 and 7 days after onset (seen in the Table S1), since the staging criteria were not unified in the SCI imaging, integrating the criteria proposed by Zalewski et al. [4] and Leys et al. [13], the majority of Gd‐MRIs were completed in late acute or early subacute phase. The presence of gadolinium enhancement (Gd+) may be attributed to secondary inflammatory reactions and disruptions in the blood–brain barrier, which can result from any damage to the central nervous system [17]. Additionally, changes in vascular dilation and hemodynamics can also contribute to the presence of Gd+ [18].

Spinal cord DWI and ADC analysis are crucial for diagnosing spinal cord injuries (SCI) [19]. Unlike the high accessibility of DWI/ADC sequences following cerebral infarction, obtaining high‐quality DWI/ADC sequences for the spinal cord is relatively challenging. Nevertheless, we found that restrictions could still be detected when the average time from symptom onset to DWI/ADC was 13 days, longer than that of the previous research [4]. All 10/13 examinations were performed after 7 days (subacute phase); the other 3 cases were completed in 3, 4, and 5 days (acute or subacute phase based on the different criteria). This may be attributed to the prolonged duration of cytotoxic edema following spinal cord lesions.

The divergent treatment approaches of SCI and ITM raise controversy about the efficacy and prognosis of corticosteroid therapy in SCI [20]. Although current evidence, including recent meta‐analyses [21, 22], indicates that the benefit of corticosteroid therapy for short‐ and long‐term motor or neurological recovery after spinal cord infarction remains debatable; however, in China, spinal DWI/ADC sequences are still not widely available; consequently, before a definitive diagnosis of SCI is established, patients may receive a short empirical course of corticosteroids, either at our center or at referring hospitals, to alleviate symptoms. Whenever steroids are considered for a patient of suspected SCI, our protocol is as follows: CT angiography or contrast‐enhanced MRI is first performed to exclude steroid‐contraindicated conditions such as spinal vascular fistulas; an empirical dose is then given, and treatment is discontinued as soon as the diagnosis of SCI is clarified via DWI/ADC sequence.

Regrettably, there is no proven effective therapy for spinal cord infarction [20]. Corticosteroids therapy does not appear to have a substantial impact on SCI, and most SCI patients in our study do not experience subjective improvement in symptoms following corticosteroids therapy. Nevertheless, during the initial phases of the illness, particularly when the presence of inflammatory myelitis cannot be definitively excluded prior to the implementation of diffusion‐weighted imaging (DWI), it is advisable to employ corticosteroids experimentally to prevent the advancement of the disease. Simultaneously, the secondary inflammatory response following SCI may justify the need for corticosteroid use.

Regarding antiplatelet medication, numerous studies have verified that individuals with spinal cord injuries frequently exhibit multiple risk factors for cardiovascular and cerebrovascular diseases. This study also verifies that over 50% of cases exhibit at least one cardiovascular and cerebrovascular risk factor. Considering that the etiology of SCI may bear resemblance to that of stroke, it is reasonable to employ antiplatelet medication. The patients included in this study primarily have a diagnosis of SCI or have been excluded from other potential causes based on clinical evidence. It is worth noting that no adverse events, such as hemorrhage, were observed during antiplatelet therapy, although the sample size was small. Nevertheless, this study sheds light on the potential of antiplatelet therapy in SCI.

The prognosis results in our study align with prior research, elucidating the prognostic attributes of SCI [23, 24]. While short‐term symptoms may be more conspicuous, long‐term monitoring indicates that most patients do not necessitate walking assistance. This aligns with the findings of the functional outcomes of 115 SCI patients [23]. The study found that the patients who had a favorable prognosis were primarily young individuals who had received steroid or antiplatelet medications during their treatment and had undergone intensive rehabilitation therapy. The rate of recurrence in ITM patients following hormone treatment is relatively low, and their dependence on the drug is also minimal.

This article has limitations. Firstly, it is retrospective research from a single center; the differences in follow‐up to the onset date may affect the interpretation of functional outcomes. Secondly, the study has a limited sample size, which may affect the generalizability of the findings, and it also restricts multivariable adjustment for assessing the discriminatory performance of individual predictors and their potential influence on prognosis. The utilization rate of Diffusion‐Weighted Imaging (DWI) is comparatively low. A prospective and multi‐center cohort needs to be completed in future.

## Author Contributions

All authors made a significant contribution to the work reported, whether that is in the conception, study design, execution, acquisition of data, analysis and interpretation, or in all these areas; took part in drafting, revising, or critically reviewing the article; gave final approval of the version to be published; have agreed on the journal to which the article has been submitted; and agree to be accountable for all aspects of the work. All authors have read and approved the final submitted manuscript.

## Funding

This work was supported by the National Natural Science Foundation of China (Grant number: 82371302 to X.Q.Z.) and Beijing Natural Science Foundation grant (JQ23027).

## Conflicts of Interest

The authors declare no conflicts of interest.

## Supporting information


**Table S1:** The time (days) and relative results of images of SCI patients. DWI/ADC, diffusion‐weighted imaging/apparent diffusion coefficient; MRI + C, gadolinium enhancement magnetic resonance imaging; MRI, magnetic resonance imaging; SCI, spinal cord infarction.
**Table S2:** MRI findings across different etiologies of SCI patients. DWI/ADC, diffusion‐weighted imaging/apparent diffusion coefficient; Gd‐MRI, gadolinium enhancement magnetic resonance imaging; MRI, magnetic resonance imaging; SCI, spinal cord infarction. Idiopathic with ASRF, Idiopathic with atherosclerotic risk factors.
**Table S3:** FDR adjusted P values, and OR with 95% CI. CI, confidence interval; FDR, false‐discovery‐rate; MRI, magnetic resonance imaging; OR, odds ratio. /: OR and 95% CI cannot be calculated for limited cases in some variables.
**Figure S1:** Flowchart of patient selection and study workflow. CTA, computed tomography angiography; DSA, digital subtraction angiography; DWI/ADC, diffusion‐weighted imaging/apparent diffusion coefficient; EMR, electric medical record; MRI, magnetic resonance imaging; SCI, spinal cord infarction.

## Data Availability

The data that support the findings of this study are available from the corresponding author upon reasonable request.
