# Supplementary material for: Spinal Cord Infarction Versus Idiopathic Transverse Myelitis: Clinical, Radiological, and Functional Insights From a Retrospective Cohort Study
Source: Ann Clin Transl Neurol. 2026 Jan 27;13(7):1388–97. doi: 10.1002/acn3.70312 (PMC13358551; doi:10.1002/acn3.70312)
Supplement: Supplementary file 1 — Table S1: The time (days) and relative results of images of SCI patients. DWI/ADC, diffusion‐weighted imaging/apparent diffusion coefficient; MRI + C, gadolinium enhancement magnetic resonance imaging; MRI, magnetic resonance imaging; SCI, spinal cord infarction. Table S2: MRI findings across different etiologies of SCI patients. DWI/ADC, diffusion‐weighted imaging/apparent diffusion coefficient; Gd‐MRI, gadolinium enhancement magnetic resonance imaging; MRI, magnetic resonance imaging; SCI, spinal cord infarction. Idiopathic with ASRF, Idiopathic with atherosclerotic risk factors. Table S3: FDR adjusted P values, and OR with 95% CI. CI, confidence interval; FDR, false‐discovery‐rate; MRI, magnetic resonance imaging; OR, odds ratio. /: OR and 95% CI cannot be calculated for limited cases in some variables. Figure S1: Flowchart of patient selection and study workflow. CTA, computed tomography angiography; DSA, digital subtraction angiography; DWI/ADC, diffusion‐weighted imaging/apparent diffusion coefficient; EMR, electric medical record; MRI, magnetic resonance imaging; SCI, spinal cord infarction. [file ACN3-13-1388-s001.docx]

Table S1. The time (days) and relative results of images of SCI patients.

| Case | Onset to MRI | Onset to MRI+C | MRI+C result | Onset to DWI/ADC | DWI/ADC result | vascular image |
| --- | --- | --- | --- | --- | --- | --- |
| case 1 | 7 | 8 | positive | 14 | positive | CTA |
| case 2 | 2 | 2 | positive | 12 | positive | DSA/CTA |
| case 3 | 2 | 2 | positive | 12 | positive | CTA |
| case 4 | 7 | 15 | positive | 10 | positive | DSA/CTA |
| case 5 | 15 | 15 | positive | / | / | CTA |
| case 6 | 14 | 15 | positive | / | / | CTA |
| case 7 | 31 | 40 | positive | / | / | CTA |
| case 8 | 2 | 2 | positive | 7 | negative | CTA |
| case 9 | 13 | 16 | positive | / | / | CTA |
| case 10 | 3 | 3 | positive | 3 | positive | CTA |
| case 11 | 2 | 2 | positive | 4 | positive | CTA |
| case 12 | 2 | 2 | positive | / | / | CTA |
| case 13 | 5 | 6 | positive | / | / | CTA |
| case 14 | 4 | 6 | positive | 5 | positive | CTA |
| case 15 | 16 | 12 | positive | / | / | CTA |
| case 16 | 0 | 0 | negative | / | / | CTA |
| case 17 | 10 | 16 | negative | / | / | CTA |
| case 18 | 3 | 6 | negative | 8 | positive | CTA |
| case 19 | 2 | 2 | positive | 15 | positive | DSA/CTA |
| case 20 | 4 | 6 | positive | 12 | positive | CTA |
| case 21 | 6 | 15 | positive | 14 | positive | DSA/CTA |
| case 22 | 12 | 15 | positive | 12 | positive | CTA |

SCI, spinal cord infarction; MRI, magnetic resonance imaging; MRI+C, gadolinium enhancement magnetic resonance imaging; DWI/ADC, diffusion-weighted imaging/apparent diffusion coefficient.

Table S2. MRI findings across different etiologies of SCI patients.

| MRI findings | VAD  n=2 | Idiopathic with ASRF, n=20 | p value |
| --- | --- | --- | --- |
| Onset to T2, days | 2.0 [2.0, 7.0] | 4.5 [2.0, 12.8] | 0.701 |
| Spinal level, n(%) |  |  | 0.585 |
| Cervical | 1 (50.0) | 5 (25.0) |  |
| Thoracic | 0 (0.0) | 9 (45.0) |  |
| Lumbar | 0 (0.0) | 1 (5.0) |  |
| Cervical+ Thoracic | 1 (50.0) | 3 (15.0) |  |
| Thoracic+ Lumbar | 0 (0.0) | 2 (10.0) |  |
| DWI/ADC restriction, n(%) | 2 (100.0) | 10 (90.9) | 0.657 |
| T2-hyperintensity patterns |  |  |  |
| Owl eyes, n(%) | 0 (0.0) | 6 (30.0) | 0.364 |
| Anterior pencil-like hyperintensity, n(%) | 2 (100.0) | 12 (60.0) | 0.262 |
| Anterior U/V, n(%) | 0 (0.0) | 1 (5.0) | 0.746 |
| Anteromedial spot, n(%) | 1 (50.00) | 7 (35.0) | 0.674 |
| Holocord, n(%) | 1 (5.0) | 0 (0.0) | 0.746 |
| Hologrey, n(%) | 0 (0.0) | 2 (10.0) | 0.639 |
| The eccentric sign, n(%) | 2 (100.0) | 15 (75.0) | 0.421 |
| Concurrent acute cerebral infarct, n(%) | 0 (0.0) | 1 (5.0) | 0.746 |
| With white matter lesions, n(%) | 0 (0.0) | 3 (15.0) | 0.556 |
| Patchy lesions/ linear lesions, n(%) | 0 (0.0) | 3 (15.0) | 0.556 |
| Lesion length, mm | 69.4 [69.4,69.4] | 60.0 [34.2,115.5] | 0.643 |
| Segment invloved | 5.0 [5.0, 5.0] | 3.0 [3.0, 3.0] | 0.045* |
| Onset to Gd-MRI, days | 2.0 [2.0, 15.0] | 6.0 [2.0, 15.0] | 0.866 |
| Gadolinium enhancement, n(%) | 2 (100.0) | 17 (85.0) | 0.556 |

SCI, spinal cord infarction; MRI, magnetic resonance imaging; Gd-MRI, gadolinium enhancement magnetic resonance imaging; DWI/ADC, diffusion-weighted imaging/apparent diffusion coefficient. Idiopathic with ASRF, Idiopathic with atherosclerotic risk factors

Table S3. FDR adjusted P values, and OR with 95% CI.

| MRI findings | OR with 95% CI | q value |
| --- | --- | --- |
| Rapid onset to nadir | 58.6 (11.88, 288.97) | p<0.001 |
| Attack frequency | 1.06 (0.73, 1.53) | 0.051 |
| Time to nadir deficit | / | p<0.001 |
| Limb palsy | / | p<0.001 |
| Sensory level | / | p<0.001 |
| Bilateral weakness | 17.33 (3.55, 84.69) | p<0.001 |
| Owl eyes | / | 0.001 |
| Anterior pencil-like hyperintensity | / | p<0.001 |
| Anterior U/V | / | 0.392 |
| Anteromedial spot | / | p<0.001 |
| Holocord | / | 0.393 |
| Hologrey | / | 0.152 |
| The eccentric sign | 31.45 (3.49, 132.04) | p<0.001 |
| Concurrent acute cerebral infarct | 1.90 (0.11, 32.01) | 1 |
| With white matter lesions | / | 0.003 |
| Patchy lesions/ linear lesions | / | p<0.001 |
| Gadolinium enhancement | 1.30(0.30, 5.64) | 1 |

MRI, magnetic resonance imaging; OR, odds ratio; CI, confidence interval; FDR, false-discovery-rate.

/: OR and 95% CI cannot be calculated for limited cases in some variables.

Figure S1. Flowchart of patient selection and study workflow.


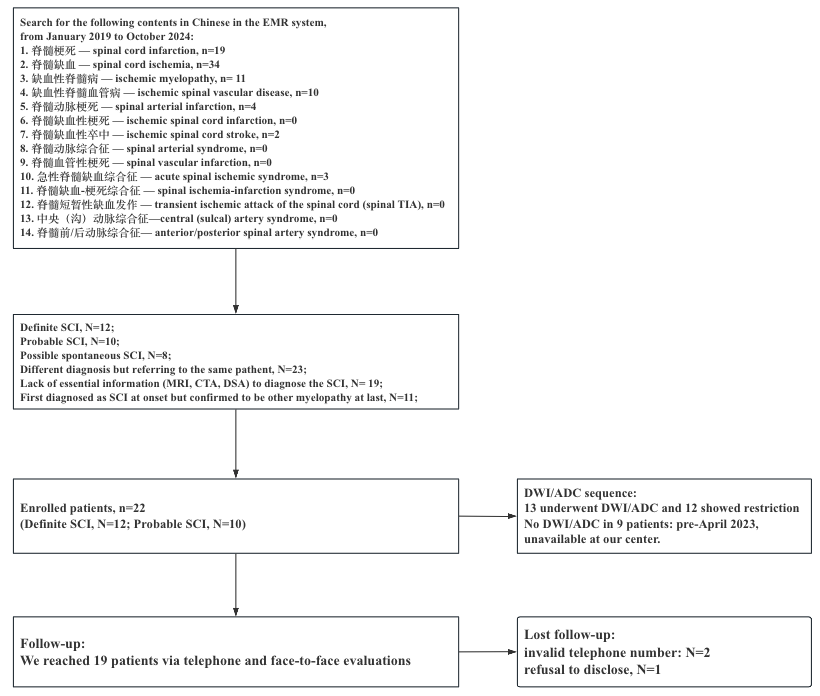


EMR, electric medical record; SCI, spinal cord infarction; MRI, magnetic resonance imaging; DWI/ADC, diffusion-weighted imaging/apparent diffusion coefficient. CTA, computed tomography angiography; DSA, Digital subtraction angiography.
